# Child HIV Exposure and CMV Seroprevalence in Botswana: No Associations With 24-Month Growth and Neurodevelopment

**DOI:** 10.1093/ofid/ofaa373

**Published:** 2020-08-22

**Authors:** Natasha O Moraka, Sikhulile Moyo, Christiana Smith, Maryanne Ibrahim, Gloria Mayondi, Jean Leidner, Kathleen M Powis, Adam R Cassidy, Betsy Kammerer, Gbolahan Ajibola, Paige L Williams, Adriana Weinberg, Rosemary Musonda, Roger Shapiro, Simani Gaseitsiwe, Shahin Lockman

**Affiliations:** 1 Research Laboratory Department, The Botswana-Harvard AIDS Institute Partnership, Gaborone, Botswana; 2 Division of Medical Virology, Department of Pathology, Stellenbosch University Tygerberg, Cape Town, South Africa; 3 Department of Immunology and Infectious Diseases, Harvard T.H. Chan School of Public Health, Boston, Massachusetts, USA; 4 Pediatric Infectious Diseases, University of Colorado Denver, Denver, Colorado, USA; 5 Department of Pediatrics, Baylor College of Medicine, Houston, Texas, USA; 6 Goodtables Data Consulting, Norman, Oklahoma, USA; 7 Department of Medicine, Massachusetts General Hospital, Boston, Massachusetts, USA; 13 Department of Paediatrics, Massachusetts General Hospital, Boston, Massachusetts, USA; 8 Department of Psychiatry, Boston Children’s Hospital, Boston, Massachusetts, USA; 9 Harvard Medical School, Boston, Massachusetts, USA; 10 Department of Biostatistics, Harvard T.H. Chan School of Public Health, Boston, Massachusetts, USA; 14 Department of Epidemiology, Harvard T.H. Chan School of Public Health, Boston, Massachusetts, USA; 11 Division of Infectious Diseases, Beth Israel Deaconess Medical Centre, Boston, Massachusetts, USA; 12 Division of Infectious Diseases, Brigham and Women’s Hospital, Boston, Massachusetts, USA

**Keywords:** anthropometrics, Botswana, children, cytomegalovirus, HIV-exposed uninfected, HIV-unexposed uninfected, neurodevelopment

## Abstract

**Background:**

We sought to identify predictors of child cytomegalovirus (CMV) infection overall and by maternal HIV status and to assess associations of child CMV status with growth and neurodevelopmental outcomes at 24 months of age in Botswana.

**Methods:**

Data and samples were used from the Botswana-based observational Tshipidi study (2010–2014), enrolling pregnant women living with and without HIV and following their infants through 2 years of age. Child plasma samples were tested at 18 months of age for anti-CMV immunoglobulin G (IgG). Associations were assessed between detectable anti-CMV IgG and growth (using the World Health Organization Child Growth Standards) and neurodevelopment (using the Bayley Scales of Infant and Toddler Development III and the Developmental Milestones Checklist) at 24 months of age.

**Results:**

Of 317 children, 215 (68%) had detectable anti-CMV IgG at 18 months of age. Comparatively, 83% (n = 178) of HIV-unexposed uninfected (HUU) children had positive CMV serology vs 47% (n = 139) of HIV-exposed uninfected (HEU) children (*P* < .01); 100% of HUU vs 10.5% of HEU children breastfed. Child CMV infection was not associated with weight-for-age, weight-for-length, or length-for-age z-scores at 24 months. In HUU children, CMV infection was associated with smaller head circumference (*P* < .01). No difference was observed by child CMV status in any neurodevelopmental domain at 24 months.

**Conclusions:**

We observed high CMV seropositivity in 18-month-old children in Botswana, with higher seropositivity among breastfed (HUU) children. Positive CMV serostatus was not associated with 24-month child growth or neurodevelopmental outcomes, with the exception of smaller head circumference among HUU CMV-positive children.

Approximately 1 in 4 infants in Southern Africa is born to a woman with HIV (WWH) [1], and the vast majority of these children remain HIV-uninfected as a result of programs to prevent vertical transmission (VT) of HIV [1, 2]. These HIV-exposed uninfected (HEU) children experience higher rates of morbidity and mortality compared with their HIV-unexposed uninfected (HUU) counterparts [2, 3], as well as poorer growth and, in some studies, poorer neurodevelopmental outcomes [4–11].

Mechanisms behind this increased susceptibility of HEU children to poor growth and developmental outcomes have not been fully elucidated. However, these outcomes may be caused by multiple factors including sequelae of antiretroviral (ARV) exposure in utero, exposure to maternal co-infections, and/or suboptimal immune responses, including to vaccinations [2, 12–14]. Some studies have identified an association between early infection with cytomegalovirus (CMV) and poorer growth and development in HEU children [15, 16]. Furthermore, a Kenyan cohort observed a 6-fold increase in the odds of CMV infection in HEU vs HUU infants, also indicating that CMV infection may lead to decreased CD8^+^ T-cell activation and impaired growth [16].

In developing countries, most adult women are CMV-seropositive (>70%), as CMV is generally acquired early in life due to breast milk transmission and crowded living conditions. In Botswana, 96% of adults with HIV are CMV immunoglobulin G (IgG) positive [17]. These high rates of preexisting CMV immunity result in a low incidence of acute CMV infection during pregnancy and thus low rates of congenital CMV infection in infants. However, some studies suggest that HEU infants are more likely than HUU infants to acquire congenital CMV due to increased risk of CMV reactivation among pregnant WWH [16, 18–23]. Congenital CMV constitutes the most common cause of nongenetic childhood hearing loss worldwide and is a significant cause of neurodevelopmental delay [24–27]. HEU infants in developing settings are also at high risk of acquiring CMV in early life [16, 22, 28], but the impact of early childhood CMV infection on growth and neurodevelopmental outcomes in this vulnerable population is unknown.

We sought to determine the association between child CMV infection at 18 months and 2-year growth and neurodevelopmental outcomes among HEU and HUU children who were followed in a prospective observational study in Botswana.

## METHODS

### Study Population

This study used existing samples and data from the “Tshipidi” study [29, 30]. Tshipidi was a prospective observational cohort study that enrolled WWH and HIV-negative women who were pregnant (88%) or within 7 days of delivery (12%) between 2010 and 2012 and followed mother–infant pairs for 2 years after delivery/birth. During the study period, WWH in Botswana with a CD4 count ≤350 cells/mL or World Health Organization (WHO) stage 3 or 4 disease were eligible for 3-drug antiretroviral treatment (ART); all others were eligible for prophylaxis with zidovudine (ZDV) during pregnancy and single-dose nevirapine (NVP) during labor and delivery. WWH chose their infant feeding method following counseling and were generally encouraged to formula-feed (and provided with free infant formula) according to Botswana government policy at the time. HIV-negative women were encouraged to breastfeed. HIV-1 RNA and CD4 count were measured at enrollment among WWH. Tshipidi study eligibility required documentation of HIV DNA polymerase chain reaction results for all HIV-exposed infants at birth, 1 month, and repeated at 6 and 12 months if the infant breastfed for any period. HIV testing was performed on all infants at premature discontinuation of study participation or 18 months of age (by enzyme-linked immunosorbent assay enzyme-linked immunosorbent assay [ELISA]), regardless of maternal HIV or feeding status. Children with HIV and their mothers were excluded from this analysis.

### Patient Consent Statement

Women provided written informed consent for study participation. Evaluation of the role of CMV infection in child outcomes was 1 of the prespecified, approved objectives of the Tshipidi study.

The Botswana Health Research Development Committee and the Office of Human Research Administration at Harvard T.H. Chan School of Public Health granted ethics approvals.

### CMV, Anthropometry, and Neurodevelopmental Testing

All children with at least 1 available stored plasma sample from the 18-month visit were tested for anti-CMV IgG using the CMV IgG ELISA Kit, Trinity Biotech. The relative sensitivity and specificity of the CMV (IgG) ELISA Kit (Trinity Biotech) that was used to test CMV in infant plasma are 99.2% and 94.1%, respectively. Child length, weight, and head circumference were measured by trained study staff in the standardized fashion. Length was measured by 2 individuals with the child in a recumbent position, with 1 person (usually the infant’s mother or caregiver) holding the child’s head at the top of an infant measuring mat and the second individual, always a trained study staff member, ensuring straight alignment of the child with extension of the legs and with the heels used for the measurement at the bottom of the mat. Infants were weighed without nappies/diapers. Trained staff measured head circumference using a nonstretch measuring tape by securely wrapping the tape around the widest possible circumference of the head. Weight-for-age, weight-for-length, length-for-age, and head circumference-for-age z-scores were calculated using WHO 2006 Childhood Growth standards, which account for a child’s age and sex [31]. Neurodevelopmental assessment was performed using the Bayley Scales of Infant and Toddler Development, Third Edition (BSIDIII). Neurodevelopmental assessments were performed by research nurses who were trained and monitored by pediatric neuropsychologists. Cognitive, receptive language, expressive language, fine motor, and gross motor domains were assessed. We also administered the Developmental Milestones Checklist (DMC), a parent report questionnaire of language, motor, and personal/social skills developed for use in Sub-Saharan Africa [32, 33]. Details of test adaptation, training of assessors, and monitoring of data quality have been described elsewhere [29].

### Statistical Analysis

We used descriptive statistics to summarize the frequency of characteristics according to child CMV serostatus at 18 months of age. We undertook univariable and multivariable logistic regression analyses to evaluate the association between potential predictors and child CMV serostatus at 18 months. Any covariate with *P* < .2 in univariable analysis was included in final multivariable analysis of predictors of positive child CMV status. The association between child CMV infection status at 18 months of age and anthropometrics at 24 months of age was analyzed using linear regression using growth as a continuous variable, adjusting for covariates selected a priori (maternal education level and infant prematurity); 2-sample *t* tests were also performed by comparing z-scores between CMV-infected and CMV-uninfected children. Neurodevelopmental test scores at 24 months of age for each of the 5 Bayley domains and DMC tests were compared by child CMV serostatus at 18 months of age using 2-sample *t* tests (separately for each domain tested). Because maternal HIV status and infant feeding method were each important potential confounders of the relationship between child CMV status and child growth and neurodevelopmental outcomes, we conducted multivariable analyses of these outcomes adjusted for feeding method and for HIV exposure status separately; these variables could not be included in the same multivariable model due to collinearity. *P* values <.05 were considered statistically significant. Stata, version 14.0, was used for all analyses.

## RESULTS

### Enrollment, Testing, and Baseline Characteristics

A total of 912 mothers and 910 of their live-born children were enrolled in the Tshipidi study: 454 WWH with 453 respective children and 458 HIV-uninfected women with 457 respective children. Plasma samples were available for 317 (35%) children at 18 months (139 HEU and 178 HUU); all of these samples were tested for CMV IgG. The characteristics of infants whose samples were tested for CMV IgG and those whose samples were not are shown in Supplementary Table 1.

Among the 317 children and their mothers who were included in this analysis, the median maternal age (interquartile range) was 29 (25–36) years. (Table 1) Among the 139 WWH, the median CD4 cell count at enrollment was 406 cells/mm^3^ and the median HIV-1 RNA was 3.2 log_10_ copies/mL. Fifty-eight (42%) WWH took 3-drug ART during pregnancy, and the remaining 58% received zidovudine prophylaxis. Fourteen (10%) WWH opted to breastfeed, compared with 178 (100%) HIV-negative mothers. The median gestational age at delivery (range) was 40 (38–41) weeks and was similar between WWH and those without HIV. The only statistically significant finding in Table 1 is that more HUU infants were likely to be CMV IgG positive at 18 months after adjusting for child sex, preterm birth, and maternal education level.

**Table 1.** Maternal and Child Baseline Characteristics by Child CMV IgG Status at 18 Months and Factors Associated With Child CMV Seropositivity in Univariable and Multivariate Analyses

	Child CMV IgG Serostatus at 18 Months of Age		Univariable Analysis		Multivariable Analysis^b^	
Characteristic	CMV Positive (n = 215), No. (%), Median (Q1, Q3), or Median (IQR)	CMV Negative (n = 102), No. (%), Median (Q1, Q3), or Median (IQR)	OR (95% CI)^a^	*P* Value	OR (95% CI)^a^	*P* Value
All mothers (n = 317)						
Maternal age, y	29 (25, 36)	31 (26, 35)	0.99 (0.9 to 1.0)	.7		
Mother HIV positive (n = 139)	66 (31 )	73 (72)	0.2 (0.1 to 0.3)	<.01	0.2 (0.1 to 0.3)	<.01
Mother employed (n = 52)	28 (13)	39 (38)	1.5 (0.7 to 3.0)	.3		
No. of people living in household	7 (4–10)	6 (4–9)	1.1 (0.98 to 1.1)	.2		
Maternal education level (n = 314)						
None or primary	17 (8)	16 (16)	1 (ref)			
Secondary	166 (77)	72 (71)	2.2 (1.0 to 4.5)	.04	1.3 (0.6 to 2.9)	.5
Tertiary	33 (15)	13 (13)	2.2 (0.8 to 5.6)	.1	0.99 (0.7 to 1.4)	.96
Child characteristics (n = 317)						
Male	104 (48)	59 (58)	1.5 (0.91 to 2.4)	.1	1.7 (1.0 to 2.9)	.05
Birth weight, kg	3.1 (2.8, 3.4)	3.1 (2.7, 3.5)	1.3 (0.8 to 1.9)	.2		
Birth height, cm	50 (49, 52)	51 (48, 52)	0.1 (0.9 to 1.0)	.7		
Birth head circumference, cm	34 (33, 35)	34 (33, 35)	0.9 (0.8 to 1.1)	.3		
Low birthweight (<2.5 kg)	20 (9)	12 (12)	0.5 (0.02 to 13.9)	.7		
Preterm birth (<37 estimated weeks of gestation)	24 (11)	5 (5)	2.4 (0.90 to 6.6)	.1	2.4 (0.8 to 7.2)	.1
Birth defects^c^						
Yes	3 (1.4)	0	NA			
No	212 (98.6)	102 (100)	NA			
Child ever breastfed						
Yes	161 (75)	31 (30)	1 (ref)			
No	54 (25)	71 (70)	6.8 (4.1 to 11.5)	<.01		

Abbreviations: CMV, cytomegalovirus; IgG, immunoglobulin G; IQR, interquartile range; OR, odds ratio; Q1, 25th percentile; Q3, 75th percentile.

^a^Odds ratio for having characteristic if CMV IgG-positive.

^b^Adjusted for child sex, preterm birth, maternal education level, and maternal HIV status. Breastfeeding was not included in the model due to collinearity with maternal HIV status.

^c^Birth defects reported: craniosynostosis (2 infants) and bilateral supranumerary digits (1 infant).

### Prevalence and Predictors of Positive CMV IgG at 18 Months of Age

Overall, 215 (68%) children tested positive for CMV IgG at 18 months. A greater proportion of HUU children had positive CMV IgG (83%) than HEU children (47%; *P* < .01). Similarly, breastfed children had higher rates of CMV seropositivity (75%) compared with those who did not breastfeed (30%; *P* < .01), (Table 1). Among the 14 WWH who breastfed, CMV-seropositive children were breastfed longer than CMV-seronegative children (median, 26 weeks vs 13 weeks, respectively; *P* = .20). A similar trend was observed for HUU children: CMV-seropositive children breastfed for a median of 55 weeks, vs 46 weeks for children who were CMV seronegative (*P* = .30). Other than breastfeeding (and negative maternal HIV status, which was highly correlated with breastfeeding), higher maternal education was the only factor associated with positive child CMV IgG in the univariable analysis, although it did not remain a significant predictor in the multivariable model. The additional socioeconomic factors available from the original study data set included cooking method, availability of electricity, cooking method, source of water; these showed no differences between the comparator groups (data not shown). Among HEU children, maternal HIV-1 RNA, CD4 cell count, and ARV regimen during pregnancy were not associated with child CMV serostatus (Table 2).

**Table 2.** Characteristics of Mothers With HIV at Enrollment, by Child CMV IgG Status at 18 Months

	Child CMV IgG Serostatus at 18 Months of Age		Univariable Analysis	
Characteristic	CMV Positive (n = 66), No. (%), Median (Q1, Q3), or Median (IQR)	CMV Negative (n = 73), No. (%), Median (Q1, Q3), or Median (IQR)	OR (95% CI)^a^	*P* Value
HIV-exposed infants (n = 139)				
Maternal baseline HIV-1 RNA (n = 122), log_10_ copies/mL	3.4 (1.6–4.2)	3.2 (1.6–4.0)	1.1 (0. to 1.5)	.5
Maternal baseline HIV-1 RNA <400 cp/mL (n = 46)	22 (33)	24 (33)	1.0 (0.99 to 1.0)	.4
Maternal baseline CD4 (n = 139), cells/mm^3^	411 (330, 546)	387 (324, 498)	1.0 (0.99 to 1.0)	.5
Type of prenatal ARVs				
ZDV (n = 81)	39 (59)	42 (57)		
3-drug ART (n = 58)	27 (41)	31 (42)	1.1 (0.5 to 2.0)	.9

Abbreviations: ARVs, antiretrovirals; ART, antiretroviral therapy; CMV, cytomegalovirus; IgG, immunoglobulin G; IQR, interquartile range; OR, odds ratio; Q1, 25th percentile; Q3, 75th percentile; ZDV, zidovudine.

^a^Odds ratio for having characteristic if CMV IgG-positive.

### Child CMV Infection Status and 24-Month Growth

Child CMV seropositivity at 18 months was not associated with weight-for-age, length-for-age, or weight-for-length z-scores at 24 months (Table 3). HUU CMV-infected children had lower head circumference-for-age z-scores at 24 months compared with HUU who were CMV-uninfected (*P* < .01) (Table 3). This association between child CMV status and head circumference-for-age z-score was not observed in HEU children. The mean head circumference was 48.1 cm (95% CI, 47.7 to 48.4) and 47.9 cm (95% CI, 47.6 to 48.1) for CMV-uninfected and CMV-infected infants, respectively. Adjusting for breastfeeding status in separate analysis (data not shown) did not show any associations between CMV and all anthropometric outcomes.

**Table 3.** Effect of Child 18-Month CMV Serostatus on Child Anthropometrics at 24 Months, Overall, and Stratified by HIV Exposure Group

Anthropometric Z scores at 24 Months	CMV Seropositive, Mean (SD)	CMV Seronegative, Mean (SD)	Regression Coefficient (95% CI)	*P* Value	Regression Coefficient^a^ (95% CI)	*P* Value^a^
All children (n = 317)						
WAZ	–0.58 (1.1)	–0.50 (1.3)	0.0004 (–0.03 to 0.03)	.98		
LAZ	–0.67 (1.4)	–0.58 (1.3)	–0.02 (–0.05 to 0.00)	.10		
WLZ	–0.35 (1.1)	–0.31 (1.3)	0.003 (–0.002 to 0.008)	.23		
HCZ	0.06 (1.2)	0.13 (1.3)	–0.01 (–0.05 to 0.03)	.6		
HEU (n = 139)						
WAZ	–0.65 (1.2)	–0.49 (1.4)	–0.02 (–0.09 to 0.06)	.66		
LAZ	–0.74 (1.4)	–0.45 (1.4)	–0.07 (–0.14 to 0.001)	.05		
WLZ	–0.40 (1.1)	–0.39 (1.3)	0.04 (–0.04 to 0.12)	.31		
HCZ	0.16 (1.2)	–0.08 (1.4)	0.03 (–0.04 to 0.10)	.38		
HUU (n = 178)						
WAZ	–0.6 (1.1)	0.5 (1.1)	–0.004 (–0.03 –0.02)	.75		
LAZ	–0.65 (1.4)	–0.85 (1.2)	–0.008 (–0.03 to 0.01)	.46		
WLZ	–0.33 (1.1)	–0.17 (1.3)	0.001 (– 0.003 to 0.006)	.53		
HCZ	0.03 (1.2)	0.73 (0.9)	0.07 (–0.11 to –0.02)	<.01	0.32 (–0.17 to 0.81)	.2

Abbreviations: CMV, cytomegalovirus; HCZ, head circumference-for-age z-score; HEU, HIV-exposed uninfected infants; HUU, HIV-unexposed uninfected infants; LAZ, length-for-age z-score; WAZ, weight-forage z-score; WLZ, weight-for-length z-score.

^a^Adjusted for maternal education level, infant prematurity.

### Child CMV Infection Status and 24-Month Neurodevelopmental Outcomes

The results of child neurodevelopmental testing at 24 months did not differ by CMV serostatus for any of the domains/tests in univariable analyses (Table 4), nor after adjusting for HIV exposure status or (separately) for breastfeeding status (data not shown).

**Table 4.** Neurodevelopmental Test Scores at 24 Months by Child CMV Serostatus at 18 Months

	No.	CMV Positive (n = 215), Mean (SD)	CMV Negative (n = 102), Mean (SD)	Unadjusted Mean Difference (95% CI)	*P* Value
Bayley III domain					
Cognitive	276	53.1 (3.3)	52.9 (3.2)	–0.2 (–1.0 to 0.6)	.6
Gross motor	263	52.8 (2.6)	52.9 (2.5)	0.1 (–0.6 to 0.7)	.8
Fine motor	279	37.2 (1.6)	37.2 (1.5)	0.03 (–0.4 to 0.4)	.9
Receptive language	274	20.6 (3.2)	20.5 (2.9)	–0.1 (– 0.9 to 0.7)	.8
Expressive language	273	25.2 (4.2)	25.1 (4.3)	–0.1 (–1.1 to 0.9)	.8
DMC domain					
Locomotor	310	32.1 (2.0)	32.3 (1.4)	0.1 (–0.3 to 0.6)	.5
Fine motor	307	19.5 (1.9)	19.4 (2.0)	–0.1 (–0.6 to 0.3)	.5
Language	310	16.3 (2.7)	16.3 (2.2)	0.04 (–0.6 to 0.6)	.9
Personal-social	310	44.7 (3.9)	44.9 (2.7)	0.2 (–0.6 to 1.1)	.6

Mean scores were compared using the paired Student *t* test.

Abbreviations: CMV, cytomegalovirus; DMC, Development Milestones Checklist.

## DISCUSSION

Approximately two-thirds of 18-month-old children in Botswana had positive CMV serology, with CMV infection strongly associated with breastfeeding. Higher proportions of children born to women without HIV were CMV-seropositive, likely due to much higher rates of breastfeeding in HUU compared with HEU children. Breastfeeding is known to be a common route of postnatal CMV infection [34, 35], with longer duration of breastfeeding associated with greater risk of CMV acquisition among HEU children (a finding that we confirmed) [15].

We found an association between child CMV infection and smaller head circumference-for-age z-scores at 24 months among HUU but not HEU children. A similar observation was made in a Zambian cohort, which showed that 18-month CMV seropositivity in children was associated with decreased head circumference as well as stunting (length-for-age z-score < –2) in both HEU and HUU children [15]. Furthermore, a Kenyan cohort found a significant negative association between CMV viral load and weight-for-age z-score and head circumference-for-age z-scores among HEU and HUU children [16]. We did not measure CMV viral loads in infants for this analysis; therefore, we are unable to determine whether active CMV disease contributed to anthropometric outcomes in our cohort. Other than head circumference, we did not observe differences in anthropometrics through 24 months of age by child CMV infection status.

Several studies have attributed poorer neurodevelopment to CMV infection and HIV exposure in children [15, 24, 26]. We did not observe significant differences in 24-month neurodevelopmental outcomes in CMV-seropositive vs -seronegative children. This finding is in contrast to the Zambian study, which observed decreased psychomotor skills in CMV-seropositive HEU, but not HUU, children at 18 months [15]. Of note, the Mental Development Index did not differ by CMV status. It is possible that this difference in findings could be related to a higher prevalence or degree of CMV viremia in pregnant WWH in the Zambian study, due to less ART coverage and hence greater immunosuppression. The Zambian study was conducted from 2005 to 2009, when ART coverage in the region was lower; it does not provide maternal CD4 count, and ART coverage is not described. In our previous analyses of Tshipidi data, neurodevelopmental outcomes were similar between HEU and HUU children, while breastfeeding was associated with better neurodevelopmental outcomes in general [29]. Furthermore, in the absence of infant CMV infection, transplacentally transferred maternal antibodies are lost by 18 months of age. Hence, the presence of CMV IgG at 18 months of life establishes that the infant is infected with CMV. This benchmark has been used in previous studies. For example, the same study in Zambia tested a total of 460 (57%) individuals for HCMV antibody at 18 months and assessed development using the Mental Development Index and Psychomotor Development Index [15].

Our study had several limitations. First, a key limitation of this study is potential selection bias due to a relatively small proportion (~1/3) of the original cohort being included in the secondary analysis. There were significant differences in those who were included and excluded, including breastfeeding and preterm birth status. However, we believe that this paper may be very beneficial as it is the first of its kind in the country. Moreover, we were not able to determine the timing of CMV infection in children, including whether infection was congenital or postpartum. We are also unable to comment on duration of exposure, which may contribute to varying results in terms of neurodevelopment. The vast majority of infected children likely acquired CMV after delivery, given that congenital CMV infection is rare in populations with high rates of maternal CMV seropositivity before pregnancy, and also given the strong association between CMV and breastfeeding in our study. In addition, our sample size was relatively small, and our assessments were only through 24 months of age and may not predict growth or neurodevelopment at older ages. Finally, it was not possible to separate the effects of HIV exposure from infant feeding status, given that all women without HIV opted to breastfeed while 90% of WWH formula-fed their infants.

In summary, we observed a high prevalence of CMV seropositivity among children in Botswana at 18 months, especially in breastfed HUU children. Postpartum CMV infection is unlikely to explain differences in growth or neurodevelopment between HEU and HUU children, especially where exclusive formula feeding from birth is practiced and where WWH are receiving ART and/or do not have advanced HIV disease. Reassuringly, for all children, CMV infection by 18 months had no apparent negative impact on growth or neurodevelopment through 24 months of age.

## Supplementary Data

Supplementary materials are available at *Open Forum Infectious Diseases* online. Consisting of data provided by the authors to benefit the reader, the posted materials are not copyedited and are the sole responsibility of the authors, so questions or comments should be addressed to the corresponding author.

ofaa373_suppl_Supplementary_Figure_S1

ofaa373_suppl_Supplementary_Table_1
