# Supplementary figures and images for: Child HIV Exposure and CMV Seroprevalence in Botswana: No Associations With 24-Month Growth and Neurodevelopment
Source: Open Forum Infect Dis. 2020 Aug 22;7(10):ofaa373. doi: 10.1093/ofid/ofaa373 (PMC7539691; doi:10.1093/ofid/ofaa373)

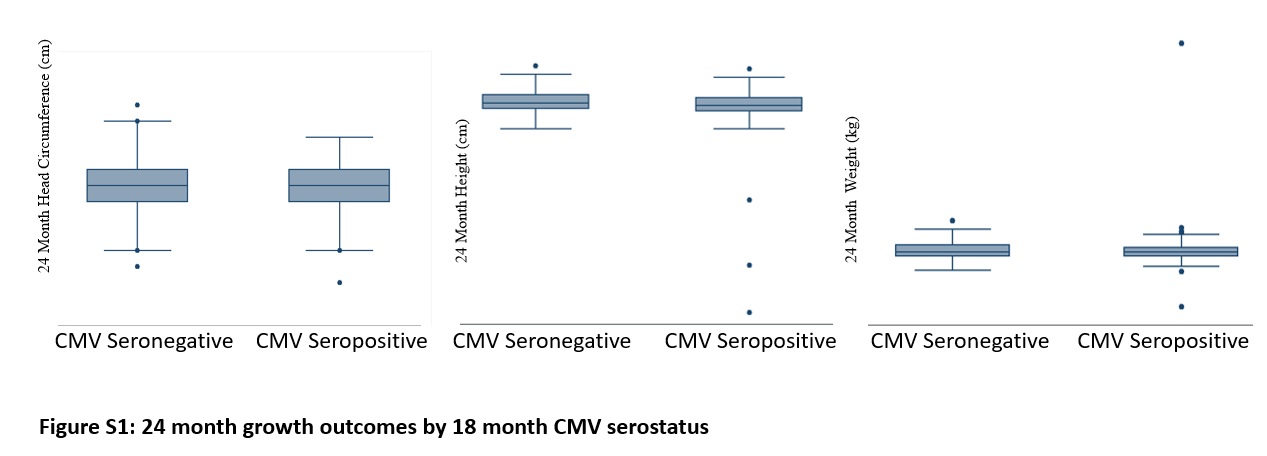

Supplement: ofaa373_suppl_Supplementary_Figure_S1 [file ofaa373_suppl_supplementary_figure_s1.jpeg]
