# Supplementary material for: Child HIV Exposure and CMV Seroprevalence in Botswana: No Associations With 24-Month Growth and Neurodevelopment
Source: Open Forum Infect Dis. 2020 Aug 22;7(10):ofaa373. doi: 10.1093/ofid/ofaa373 (PMC7539691; doi:10.1093/ofid/ofaa373)
Supplement: ofaa373_suppl_Supplementary_Table_1 [file ofaa373_suppl_supplementary_table_1.docx]

**SUPPLEMENTAL TABLES**

**Table S1: Maternal baseline characteristics by child CMV IgG testing at 18 months**

|  | Child CMV IgG tested at 18 months of age | |  | |
| --- | --- | --- | --- | --- |
| Characteristic | **YES**  **(n= 317)** | **NO**  **(n= 593)** | | **P value** |
| *All mothers (n =317)* |  |  | |  |
| Median maternal age in years (Q1, Q3) | 30 (24, 35) | 29 (24, 33) | | 0.1 |
| Mother HIV positive (n = 453) | 139 (44%) | 314 (53%) | | 0.01^a^ |
| Mother Employed (n = 149) | 52 (16%) | 97 (16%) | | 0.9^a^ |
| Median maternal baseline HIV-1 RNA (log_10_ copies/mL, IQR ) ,  n = 344 | 3.2 (1.6 , 4.0) | 3.1 (2.6 ,4.0) | | 0.7 |
| Type of prenatal ARVs |  |  | |  |
| ZDV (n =280 ) | 81 (26%) | 199 (34%) | |  |
| 3-drug ART (n =136) | 58 (18%) | 78 (13%) | | 0.01^a^ |
| Child characteristics (n = 451) |  |  | |  |
| Male | 163(51%) | 288 (48%) | | 0.4^a^ |
| Low birthweight (<2.5 kg) | 25 (7.9%) | 43 (7.2%) | | 0.8 |
| Median Birthweight in Kg (Q1, Q3) |  |  | |  |
| Male | 3.2 (2.9, 3.5) | 3.1 (2.8, 3.5) | | 0.3^a^ |
| Female | 3.0 (2.7, 3.3) | 3.0 (2.7, 3.4) | | 0.6^a^ |
| Median Birth length in cm (Q1, Q3) |  |  | |  |
| Male | 51 (49, 53) | 50 (49,52) | | 0.1^a^ |
| Female | 50 (48,52) | 50 (48,52) | | 0.2^a^ |
| Median birth head circumference in cm (Q1, Q3) |  |  | |  |
| Male | 35 (34, 36) | 35 (34, 36) | | 0.1^a^ |
| Female | 34 (33, 35) | 34 (33, 35) | | 0.07^a^ |
| Preterm birth (<37 estimated weeks gestation) | 29 (9.1%) | 99 (17%) | | <0.01^a^ |
| Birth Defects (n = 15)^b^ |  |  | |  |
| Yes | 3 (0.9%) | 12 (2.0%) | | 0.2^a^ |
| Child Ever Breastfed (n = 487) |  |  | |  |
| Yes | 192(61%) | 295 (50%) | | 0.01^a^ |

^a^ P-values calculated using Wilcoxon Rank sum Test

CI, confidence interval; CMV, Cytomegalovirus; HAART, highly active antiretroviral therapy; HIV, human immunodeficiency virus; IQR, interquartile range; OR, odds ratio; Q1, 25th percentile; Q3, 75th percentile; SD, standard deviation; ZDV, zidovudine

b Birth Defects reported included: anencephaly (2 infants) , craniosynostosis (8 infants) , pyloric stenosis (1 infant) , other gastrointestinal (GI) anomalies (1 infant) , other eye abnormally (2 infants) and defects classified as other defect/syndrome (1 infant)
